# RECIST 1.1 and serum thyroglobulin measurements in the evaluation of responses to sorafenib in patients with radioactive iodine-refractory differentiated thyroid carcinoma

**DOI:** 10.3892/ol.2013.1424

**Published:** 2013-06-25

**Authors:** MAOMEI RUAN, YAN SHEN, LIBO CHEN, MINGHUA LI

**Affiliations:** 1 Department of Nuclear Medicine, Shanghai Sixth People’s Hospital, Shanghai Jiao Tong University, Shanghai 200233;; 2 Department of Radiology, Shanghai Chest Hospital, Shanghai Jiao Tong University, Shanghai 200030;; 3 Institute of Interventional and Diagnostic Radiology, Shanghai Sixth People’s Hospital, Shanghai Jiao Tong University, Shanghai 200233, P.R. China

**Keywords:** differentiated thyroid carcinoma, thyroglobulin, RECIST, molecular targeted therapy, sorafenib

## Abstract

The present study was designed to investigate the association between response evaluation criteria in solid tumors (RECIST) 1.1 and 1.0, and to explore the utility of thyroglobulin (Tg) measurements in assessing tumor responses to sorafenib in patients with radioactive iodine (RAI)-refractory differentiated thyroid carcinoma (DTC). In total, 23 patients with RAI-refractory DTC were enrolled. A comparison of RECIST 1.1 and 1.0 was performed in all patients with measurable disease. Following the exclusion of patients who were positive for anti-Tg antibody, the correlation between RECIST 1.1 and Tg was investigated in patients with measurable disease, and the concordance of the change in Tg between these patients and the patients with non-measurable disease only was analyzed over time. Tumor responses, assessed by RECIST 1.1 and 1.0, were concordant in 96% of the 23 records. However, the number of target lesions, according to RECIST 1.1, was significantly lower than when using RECIST 1.0. Progressive disease (PD) was identified in one of the five patients who underwent fluorodeoxyglucose-positron emission tomography (FDG-PET)/computed tomography (CT) scanning. A correlation between the Tg levels and the sum of the diameters of the target lesions was verified, with the percentage decrease in Tg levels significantly greater than that in the radiograph, demonstrating shrinkage. Furthermore, the percentage change in Tg levels was consistent between the patients with measurable disease and the subjects with non-measurable disease only. In conclusion, in patients with RAI-refractory DTC, RECIST 1.1 is highly concordant with RECIST 1.0 in the assessment of responses to sorafenib treatment, with the advantage of simplified procedures and the complementary use of FDG-PET. Tg measurements, in concordance with RECIST 1.1, are valuable in the evaluation of tumor responses.

## Introduction

The incidence of differentiated thyroid carcinoma (DTC) has increased worldwide over the past two decades, with papillary thyroid carcinoma (PTC) being markedly more common than follicular thyroid carcinoma (FTC) (1,2). The prognosis of DTC is generally favorable due to the indolent nature of the disease and the efficacy of combined treatment comprising surgery, radioactive iodine (RAI) and levothyroxine. However, 10–20% of patients with DTC develop distant metastases, approximately half of which do not respond to traditional therapies. In RAI-refractory DTC patients, there is no standard therapy and the 10-year survival rate has decreased to 10% (3,4).

The recent expansion of knowledge in molecular oncology has facilitated the development of targeted agents for the treatment of various types of advanced thyroid carcinoma (5). Of these agents, tyrosine kinase inhibitors (TKIs) have emerged as novel cancer therapies with promising results (6). Sorafenib is an oral, small-molecule TKI, which targets vascular endothelial growth factor receptors (VEGFRs), rearranged during transfection (RET)/PTC proteins and BRAF-mediated events (7). Four phase II trials with sorafenib have been conducted at a dose of 400 mg, twice daily, demonstrating the clinical potential and acceptable safety of the agent (8–11). We have also successfully performed two studies on sorafenib therapy for pulmonary metastases from PTC and brain metastasis from FTC, using a low-dose strategy (200 mg, twice daily) in which tolerance to the drug and a potential therapeutic effect were demonstrated in patients with RAI-refractory DTC (12,13).

To assess the objective response to molecular targeted therapy, response evaluation criteria in solid tumors (RECIST 1.0) is commonly used (14). However, a number of questions and issues have arisen with regard to RECIST 1.0, leading to a revised version (RECIST 1.1) (15–18). Recently, RECIST 1.1 has been successfully used to evaluate responses to treatment in numerous types of solid tumors, including advanced non-small cell lung cancer and advanced gastric cancer, demonstrating superiority to the original guidelines (19,20). More recently, in a study by Marotta *et al*, this novel system has also been utilized in the initial evaluation of tumor responses to sorafenib treatment in advanced RAI-refractory DTC (21). However, RECIST 1.1 has not yet been compared with RECIST 1.0 in the evaluation of tumor responses to molecular targeted therapy in patients with RAI-refractory DTC. Moreover, in this novel system, subcentimeter-sized lesions and blastic bone lesions are considered to be non-measurable. In addition, the cavitation of lesions with internal necrosis without a change in the size of the lesion, but with a paradoxical increase in the tumor size, in response to therapy due to hemorrhage or necrosis, is not able to be correctly evaluated (22,23). Although it would be ideal to have objective criteria to apply to non-measurable lesions, the very nature of the disease makes it impossible to do so (18). Therefore, quantitative strategies are required for the evaluation of the tumor response in patients with non-measurable disease only.

Serum thyroglobulin (Tg), a specific biological marker for DTC, is measured routinely and automatically in the follow-up of patients with DTC, and serves as an indicator of the efficacy of surgery and RAI therapy (24–26). A decrease in serum Tg levels following sorafenib therapy at various doses has been observed in patients with RAI-refractory DTC in a number of studies, including a previous study by our group (9,11,12). However, in evaluating responses to molecular targeted therapy, limited data with regard to the correlation between Tg levels and the radiographic response are available, while data on the role of serum Tg measurements are controversial (9,11).

Therefore, the present study was conducted to investigate the association between RECIST 1.0 and 1.1, and the correlation between serum Tg levels and the radiographic response in sorafenib-treated patients with RAI-refractory DTC and measurable disease. The feasibility of using Tg measurements in assessing the tumor responses to sorafenib treatment in patients with measurable disease and subjects with non-measurable disease only was also explored.

## Patients and methods

### Patients

Patients with RAI-refractory DTC who demonstrated evidence of disease progression within 12 months prior to the initiation of treatment, despite the administration of sufficient thyroid hormones to reduce the serum thyroid stimulating hormone (TSH) levels to <0.1 mIU/l, were enrolled in the study. Other eligibility criteria included an Eastern Cooperative Oncology Group performance status of less than two, with preserved renal, hepatic and bone marrow function. Premenopausal women were required to have negative pregnancy test results, and all patients of child-bearing age were required to use contraception. The open-label use of sorafenib was administered at a dose of 200 mg orally, twice a day. Screening evaluations, including medical history, demography, review of prior treatment, physical examination and laboratory evaluations, were completed within one week prior to sorafenib treatment initiation.

Patients were observed at four-week intervals following the initiation of treatment. At each visit, a history was taken, a physical examination was performed and complete blood count (CBC), chemistry panel and TSH, Tg and anti-Tg antibody (TgAb) levels were measured. The patients were assessed for the appearance of novel symptoms, the compliance with study medications (pill count) and concomitant medications. The response was assessed radiographically at 12-week intervals.

Approval of the protocol was received from the ethics board of Shanghai Sixth People’s Hospital prior to the initiation of the study. All subjects provided written informed consent for participation in the study.

### Laboratory studies and radiographic assessments

Serum TSH, Tg and TgAb levels were measured using a chemiluminescent immunoassay system (Immulite, Diagnostic Products Corp., Los Angeles, CA, USA). RECIST 1.0 and 1.1 were used to assess the tumor responses to sorafenib treatment.

The objective response to treatment at the baseline and at each follow-up computed tomography (CT) examination, according to the original RECIST 1.0 criteria, was assessed by a study-designated radiologist (14). Following completion of the study, tumor lesions were reviewed by the radiologist for a second time, to generate a second set of CT tumor measurements that met the RECIST 1.1 guidelines. Compared with RECIST 1.0, there were certain changes according to RECIST 1.1: Pathological lymph nodes with a short axis ≥10 and <15 mm were considered to be non-measurable lesions; and lytic bone lesions or mixed lytic-blastic lesions with identifiable soft tissue components that may be evaluated by cross-sectional imaging techniques, such as CT or magnetic resonance imaging (MRI), and cystic lesions considered to represent cystic metastases, were considered as measurable lesions (provided that they met the definition of measurability) (18). In addition, the target lesions recorded in the original measurements were reassessed if they met the criteria of RECIST 1.1. Lymph nodes with a short axis of <15 mm were excluded from the target lesions, and when the number of target lesions exceeded the limits according to RECIST 1.1 (up to five in total and up to two per organ), smaller lesions were eliminated from the target lesions. Furthermore, short-axis measurements were used for lymph nodes, as opposed to long-axis measurements. Additionally, bone lesions, which were either lytic or mixed lytic-blastic, with a soft tissue component that met the criteria for measurability were selected as target lesions. Moreover, the fluorodeoxyglucose-positron emission tomography (FDG-PET)/CT clinical reports were also reviewed for the patients who underwent such examinations during treatment, to determine whether any new lesions were detected in the FDG-PET/CT scans that met the RECIST 1.1 criteria for progression.

### Statistical analysis

All statistical analyses were performed using a statistical software program (SPSS, version 11.0; SPSS, Inc. Chicago, IL, USA). A paired Student’s t-test and a linear correlation were used to assess the differences and the correlation between RECIST 1.0 and 1.1, respectively. A rank correlation and Wilcoxon signed rank sum test were used to assess the correlation and the percentage changes between Tg levels and RECIST 1.1, respectively. An independent samples t-test and a Wilcoxon rank sum test were used to assess the changes in the Tg levels over time and the concordance of Tg levels between patients with measurable disease and non-measurable disease only, respectively. P<0.05 was considered to indicate a statistically significant difference.

## Results

### Patients

Between August, 2009 and July, 2012, 23 consecutive DTC patients, including 14 patients with RECIST-measurable disease and nine patients with non-measurable disease only (14 females, nine males; age range, 33–75 years; mean age, 54 years), who were considered to have progressive metastases resistant to RAI treatment, were enrolled in the study. None of these patients had received chemotherapy or other kinase inhibitors prior to the administration of sorafenib.

The baseline characteristics of the patients entered into the study are listed in Table I. All patients exhibited lymph node metastases, while 22 presented with lung metastases, two with bone metastases and one with brain metastases. In five of the patients with measurable disease who underwent FDG-PET/CT, uptake of FDG prior to treatment was observed. The average duration of therapy was 12 months (range, 3–25 months).

### Comparison between RECIST 1.0 and 1.1 in patients with measurable disease

**Figure 1.**
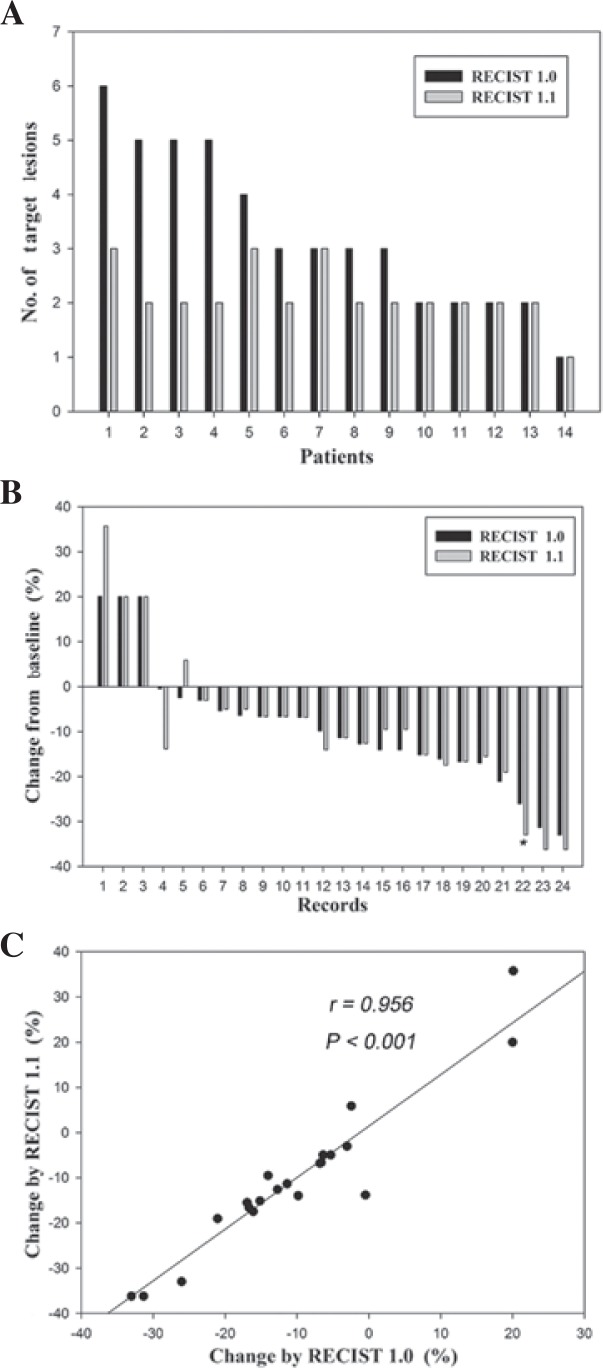
Comparison between response evaluation criteria in solid tumors (RECIST) 1.0 and 1.1 in patients with measurable disease. (A) Number of target lesions according to RECIST 1.1 compared with when using RECIST 1.0. The target lesion number according to RECIST 1.1 was significantly less than that according to RECIST 1.0 [paired Student’s t-test; P=0.006; 95% CI, 0.40–1.89]. (B) Percentage changes in the sum of the diameters of the target lesions according to RECIST 1.1 and 1.0 at all time points during therapy. Response assessments of target lesions by RECIST 1.1 and 1.0 were concordant in 23 (96%) of 24 records and discordant in one record (4%), as indicated by the asterisk (paired Student’s t-test; P=0.868; 95% CI, −2.4130–2.0497). (C) Percentage changes in the sum of the diameters of the target lesions according to RECIST 1.1 compared with that according to RECIST 1.0, at all time points during therapy. A strong positive correlation was observed between RECIST 1.1 and 1.0 (linear correlation; r=0.956; P<0.001).

Of the 23 total patients, 14 patients with measurable disease were enrolled to compare the radiographic responses to sorafenib by RECIST 1.0 and 1.1. The target lesion number, according to RECIST 1.1, was significantly lower than that using RECIST 1.0 [Fig. 1A; paired Student’s t-test; P=0.006; 95% CI, 0.40–1.89], with a decrease in eight patients (57%) and no change in the remaining six patients (43%). The number of target lesions was decreased as a result of the reduction in the number of lesions required to assess the tumor burden (from a maximum of 10 to a maximum of five in total, and from five to two per organ) for five patients. This, in turn, was as a result of the new definition of measurability of malignant lymph nodes at the baseline (a lymph node is required to have a short axis of 15 mm to be considered pathologically enlarged and measurable) for one patient, and due to the new definition of measurability of malignant lymph nodes at the baseline and the reduction in the number of lesions required to assess the tumor burden, for two patients. In one patient, the number of target lesions did not change as a result of the reduction in the number of pulmonary metastases, which was equivalent to the increase in the number of bone lesions with a soft tissue component.

Of the 14 patients with measurable disease, a total of 24 records of the percentage changes in the sum of the diameters of the target lesions at all time points during therapy were assessed by RECIST 1.0 and 1.1, respectively. Twenty-three (96%) of the 24 records demonstrated a concordant radiographic response (Fig. 1B; paired Student’s t-test; P=0.868; 95% CI, −2.4130 to 2.0497). The one discordant percentage change measurement exhibited a 26% decrease in stable disease (SD), according to RECIST 1.0, and a 33% decrease in partial response (PR), according to RECIST 1.1 (Fig. 1B). The percentage changes in the sum of the tumor diameters of the target lesions at all time points according to RECIST 1.1 and 1.0 demonstrated a high correlation (Fig. 1C; linear correlation; r=0.956; P<0.001).

**Figure 2.**
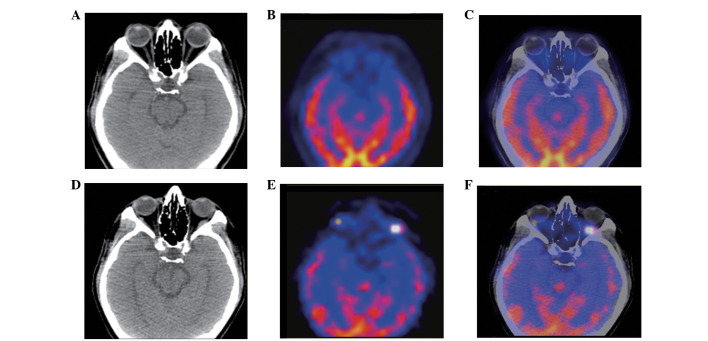
Lateral rectus lesion in the left extra-ocular muscles demonstrated by (A and D) computed tomography (CT), (B and E) Fluorodeoxyglucose-positron emission tomography (FDG-PET) and (C and F) the fusion of CT and FDG-PET. (A–C) Normal appearance of the pretreatment examination. (D–F) Twenty-four weeks after the initiation of treatment with sorafenib, a new left lateral rectus lesion was evident in the follow-up examination. This resulted in an objective response of progressive disease (PD) according to response evaluation criteria in solid tumors (RECIST) 1.1. (E) The FDG-PET image reveals a lesion with significant metabolic activity, with mean and maximum standardized uptake values of 4.8 and 10.0, respectively. (D) The CT image and (F) the fusion image verified the FDG-PET findings, showing the same leison with a dimension of 1.2×0.6 cm and a CT value of 42 Hounsfield units (HU).

The best response assessed by RECIST 1.1 had an objective PR of 14% (2/14), SD of 64% (9/14) and progressive disease (PD) of 22% (3/14), which were similar to those recorded according to RECIST 1.0 (PR, 7%; SD, 71%; and PD, 22%). The results remained the same in 13 patients (93%), while a difference was only observed in one patient (7%), as mentioned previously. Of the five patients who underwent FDG-PET/CT scanning at the baseline, PD was identified in one patient with a new lateral rectus lesion, which was revealed by the follow-up FDG-PET/CT study (Fig. 2).

### Correlation between Tg levels and the radiographic response in patients with measurable disease

**Figure 3.**
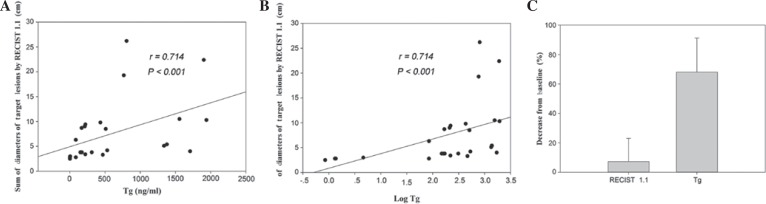
Correlation between tumor size and thyroglobulin (Tg) level in patients with measurable disease at all time points during therapy. (A) The levels of serum Tg are correlated with the sum of diameters of the target lesions, as assessed by response evaluation criteria in solid tumors (RECIST) 1.1 (rank correlation; rs=0.714; P<0.001). (B) The log of the level of Tg is also correlated with the sum of the diameters of the target lesions, as assessed by RECIST 1.1, with the same correlation coefficient (rank correlation; rs=0.714; P<0.001). (C) The percentage change in Tg levels was significantly greater than that of the radiographic response using RECIST 1.1 (Wilcoxon signed rank sum test; P<0.001).

Following the exclusion of five serum TgAb-positive patients, nine patients with measurable disease and TSH-suppressed Tg (at all time points during therapy) were enrolled to explore the correlation between Tg levels and the tumor size (as demonstrated radiographically). The levels of Tg, as well as the log of the Tg levels, were correlated with the sum of the diameters of the target lesions, as assessed by RECIST 1.1, with the same correlation coefficient (Fig. 3A and B; rank correlation; rs=0.714; P<0.001). Furthermore, the percentage change in Tg levels (mean, 68%; standard deviation, 23%) was significantly greater than that of the radiographic response (mean, 7%; standard deviation, 16%; Fig. 3C; Wilcoxon signed rank sum test; P<0.001). However, the percentage change in Tg concentration was not correlated with the change in the sum of the tumor diameters of the target lesions (rank correlation; P=0.663).

### Concordance of changes in serum Tg levels between patients with measurable and non-measurable disease

**Figure 4.**
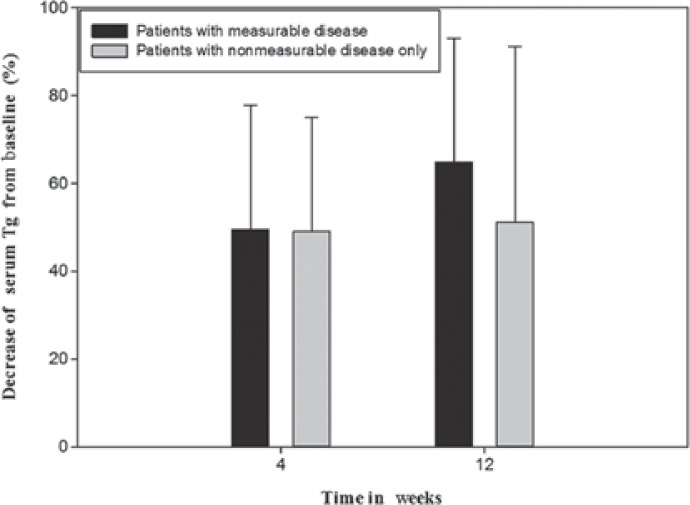
Thyroglobulin (Tg) level in nine patients with measurable disease and nine patients with non-measurable disease only recorded at the baseline and at 4 and 12 weeks following the initiation of therapy. The percentage change from the baseline in serum Tg levels for patients with measurable target lesions was consistent with that for patients with non-measurable disease only at 4 weeks (independent samples t-test; P=0.969; 95% CI, −26.67–27.69) and 12 weeks (Wilcoxon rank sum test; P=0.453).

Following the exclusion of five TgAb-positive patients from the 23-patient total, the serum Tg levels demonstrated no significant difference at the baseline between nine patients with measurable disease and nine patients with non-measurable disease only, which could not be quantitatively assessed by RECIST (Wilcoxon rank sum test; P=0.085). In addition, no significant difference in the serum Tg levels was demonstrated between these two groups of nine patients at 4 weeks (independent samples t-test; P=0.055) and 12 weeks (Wilcoxon rank sum test; P=0.122) following the initiation of treatment. Furthermore, there was no significant difference in the percentage change in serum Tg levels between patients with measurable disease (mean, 50%; SD, 28%) and non-measurable disease only (mean, 49%; SD, 26%) at 4 weeks from the baseline (Fig. 4; independent samples t-test; P=0.969; 95% CI, 26.67–27.69). The percentage change in serum Tg levels in patients with measurable disease (mean, 65%; SD, 28%) was also statistically consistent with that of patients with non-measurable disease only (mean, 52%; SD, 40%) at 12 weeks from the baseline (Fig. 4; Wilcoxon rank sum test; P=0.453).

## Discussion

In cancer therapy, a reliable assessment of the responses to treatment is essential, as the response parameters often represent surrogate markers for improved survival. For this reason, RECIST was developed and has become the main evaluation system used in current oncological investigations. However, a number of questions and issues have arisen with regard to the system, and continuous updating of RECIST is required (15–18,22,23).

In the present study, despite the significantly decreased number of target lesions assessed by RECIST 1.1, a high concordance was demonstrated between RECIST 1.1 and 1.0 in the assessment of the tumor response, indicating an almost complete agreement between the two versions. The tumor response assessed by RECIST 1.1 and 1.0 was discordant in only one record, as a result of the reduction in the number of target lesions required to assess the tumor burden. This suggested that if the value of the tumor response assessed by RECIST approached the critical value, a reduction in the number of target lesions may have resulted in a different tumor response being observed between RECIST 1.0 and 1.1, particularly in patients with small target lesions at the baseline. A reduction in the maximum number of target lesions occurred in approximately half of the patients (57%) when RECIST 1.1 was used, implying a substantial decrease in the time and effort demanded from the radiologists with this version of RECIST. An additional reason for the decrease in the number of target lesions was the new definition of measurability of malignant lymph nodes, which affected three patients via a reduction in the target lesion number and an increase in the number of non-measurable lesions. Notably, the number of target lesions in one patient did not change as a result of the reduction in pulmonary metastases, which was equivalent to the increase in bone lesions with a soft tissue component. This implied that the new definition of measurability of lytic bone lesions or mixed lytic-blastic lesions with identifiable soft tissue components resulted in an increase in the target lesion number and influenced the eligibility of this system for clinical trials.

In the present study, one patient demonstrated PD with negative FDG-PET/CT at the baseline and positive findings 24 weeks after the initiation of treatment with sorafenib, implying that it is occasionally acceptable to incorporate the use of whole-body FDG-PET scanning to complement the CT examination in the assessment of progression, as identified by RECIST 1.1. Although there were only five patients with measurable disease who underwent FDG-PET/CT examination in the study, all five exhibited positive FDG uptake, which was similar to the results demonstrated by other studies (8,21). These results confirmed the highly malignant nature of RAI-refractory DTC. Moreover, as demonstrated by Marotta *et al*, an FDG-PET assessment at the baseline may predict the radiological response, and an early FDG-PET follow-up scan may be useful for clinicians, as it may allow for the identification of patients who are unlikely to exhibit a morphological response (21). Larger and randomized studies are required to confirm the efficacy of FDG-PET/CT in the management of RAI-refractory DTC.

Despite the significant revisions made in RECIST 1.1, numerous issues remain to be resolved in the assessment of tumor responses in clinical practice. Subcentimeter-sized lesions, such as the miliary pulmonary metastases in the majority of patients with RAI-refractory DTC, are considered to be non-measurable by RECIST 1.1 criteria, resulting in difficulties in the quantitation of the tumor burden and response (18). Furthermore, as has been identified by our group and others previously, treatment with TKIs may result in the cavitation of lesions with internal necrosis without a change in lesion size, which is a challenge for radiologists who aim to obtain the measurement that best represents the tumor burden (13,22). In addition, it also important to understand that radiological lesion size results may vary due to a number of factors, including scan quality, timing of contrast administration and the identity of the interpreting radiologist (18,27). This leads to a requirement for newer methods for precisely ascertaining the tumor response, which are not solely based on the diameter, in patients receiving targeted therapy.

As a specific tumor marker for DTC, the level of serum Tg, during thyroid hormone treatment and following TSH stimulation, is correlated with the quantity of neoplastic thyroid tissue (28,29). As was demonstrated by the present study, the level of Tg and the log of the level of Tg were correlated with the sum of the diameters of the target lesions, as assessed by RECIST 1.1, with the same correlation coefficient at all time points, including the baseline and time points during the treatment. Additionally, it has been demonstrated that baseline Tg levels and Tg responses to treatment may be useful for predicting the morphological response and clinical outcome (21). However, a correlation between the change in serum Tg levels and the radiographic response was not observed in the present study, which was possibly due to the small sample size, as well as the definition of the objective response based on RECIST (9). In addition, it has been proposed that the tumor burden may be more sensitive and reproducible when measured by the tumor volume, rather than the sum of the diameters of the target lesions. Therefore, response assessments based on tumor volumes may have a positive impact on patient management and clinical trials (30).

Hoftijzer *et al* (10) demonstrated that the median time of the nadir of Tg levels was 3 months, while a rapid decrease in the serum Tg levels of 50% within 4 weeks, followed by a continued decrease in such levels (with a mean decrease of 65%) within 12 weeks of the initiation of treatment, were observed in the present study. Furthermore, the percentage change in Tg levels was significantly greater than that in the radiographic response. These results demonstrated a more marked tumor response to targeted therapy when Tg was used as an evaluation criterion compared with RECIST. This may be explained by the cytostatic effect of novel anticancer agents, which may not have reduced the tumor size significantly.

Until recently, no other quantitative criteria for assessing tumor responses to sorafenib therapy in patients with non-measurable disease only were available; the phase II (8–11) and ongoing phase III DECISION trials (31) were conducted in patients with measurable disease. In the present study, patients with measurable disease and non-measurable disease only were enrolled to evaluate the effectiveness of sorafenib treatment. Patients with non-measurable disease only were analyzed as an individual group for the first time. The levels of serum Tg between patients with measurable target lesions and patients with non-measurable disease only demonstrated no statistically significant difference at the baseline or at 4 or 12 weeks following the initiation of treatment. Furthermore, the percentage change in serum Tg levels from the baseline for patients with measurable disease was consistent with that for patients with non-measurable disease only at 4 and 12 weeks. These results suggested that such treatment in patients with non-measurable disease only exhibited a similar efficacy in patients with measurable disease. Additionally, these results demonstrated that all patients suffered from the same disease and that it was only our measurement convention that made them different. As a correlation between Tg and the sum of the diameters of the target lesions in patients with measurable disease was demonstrated, the level of Tg may potentially be used to assess the treatment response in patients with measurable disease and non-measurable disease only.

However, certain issues remain to be resolved with regard to the measurement of serum Tg. Tumor lysis during treatment with sorafenib may lead to elevated Tg levels, which may be due to either the tumor lysis itself or increased Tg synthesis (10). Furthermore, it has been demonstrated that the secretion of Tg is likely to be affected by alterations in cell signaling caused by sorafenib (8). Therefore, changes in the serum Tg level in RAI-refractory DTC treated with sorafenib require cautious interpretation. In addition, we acknowledge that the present study possessed certain limitations, including its retrospective nature, the small sample size and the short follow-up time.

In patients with RAI-refractory DTC, RECIST 1.1 demonstrated high levels of concordance with RECIST 1.0 in the assessment of responses to sorafenib therapy, with the advantage of simplified procedures and the complementary use of FDG-PET. The level of serum Tg significantly correlated with the sum of the diameters of target lesions, and the Tg response was significantly greater than the radiographic response. In addition, the percentage change in Tg levels was consistent between patients with measurable disease and subjects with non-measurable disease only. In accordance with RECIST 1.1, Tg measurements are of value in assessing the tumor response to sorafenib therapy in patients with RAI-refractory DTC, particularly in those with non-measurable disease only, for which no quantitative criteria exist.

## Figures and Tables

**Table I.** Baseline characteristics of RAI-refractory DTC patients.

Characteristics	No. of patients	%
Gender		
Female	14	60.87
Male	9	39.13
Age, years		
Mean	54	
Range	33–75	
Thyroid cancer subtype		
Papillary	22	95.65
Follicular	1	4.35
Site of Metastasis		
Lymph node	23	100.00
Lung	22	95.65
Bone	2	8.70
Brain	1	4.35
Measurability of lesions		
Measurable	14	60.87
Non-measurable	9	39.13
Prior FDG-PET/CT		
FDG-PET/CT scan completed	5	21.74
FDG uptake-positive	5	21.74
Median duration of therapy, months	10	
Average	12	
Range	3–25	

RAI, radioactive iodine; DTC, differentiated thyroid carcinoma; FDG-PET, fluorodeoxyglucose positron emission tomography; CT, computed tomography.

## References

[1] Aschebrook-Kilfoy B, Ward MH, Sabra MM, Devesa SS (2011). Thyroid cancer incidence patterns in the United States by histologic type, 1992–2006. Thyroid.

[2] Sherman SI (2003). Thyroid carcinoma. Lancet.

[3] Shaha AR, Shah JP, Loree TR (1996). Patterns of nodal and distant metastasis based on histologic varieties in differentiated carcinoma of the thyroid. Am J Surg.

[4] Durante C, Haddy N, Baudin E (2006). Long-term outcome of 444 patients with distant metastases from papillary and follicular thyroid carcinoma: benefits and limits of radioiodine therapy. J Clin Endocrinol Metab.

[5] Deshpande HA, Gettinger SN, Sosa JA (2008). Novel chemotherapy options for advanced thyroid tumors: small molecules offer great hope. Curr Opin Oncol.

[6] Zhang J, Yang PL, Gray NS (2009). Targeting cancer with small molecule kinase inhibitors. Nat Rev Cancer.

[7] Wilhelm SM, Carter C, Tang L (2004). BAY 43-9006 exhibits broad spectrum oral antitumor activity and targets the RAF/MEK/ERK pathway and receptor tyrosine kinases involved in tumor progression and angiogenesis. Cancer Res.

[8] Gupta-Abramson V, Troxel AB, Nellore A (2008). Phase II trial of sorafenib in advanced thyroid cancer. J Clin Oncol.

[9] Kloos RT, Ringel MD, Knopp MV (2009). Phase II trial of sorafenib in metastatic thyroid cancer. J Clin Oncol.

[10] Hoftijzer H, Heemstra KA, Morreau H (2009). Beneficial effects of sorafenib on tumor progression, but not on radioiodine uptake, in patients with differentiated thyroid carcinoma. Eur J Endocrinol.

[11] Cabanillas ME, Waguespack SG, Bronstein Y (2010). Treatment with tyrosine kinase inhibitors for patients with differentiated thyroid cancer: the M. D. Anderson experience. J Clin Endocrinol Metab.

[12] Chen L, Shen Y, Luo Q, Yu Y, Lu H, Zhu R (2011). Response to sorafenib at a low dose in patients with radioiodine-refractory pulmonary metastases from papillary thyroid carcinoma. Thyroid.

[13] Shen Y, Ruan M, Luo Q (2012). Brain metastasis from follicular thyroid carcinoma: treatment with sorafenib. Thyroid.

[14] Therasse P, Arbuck SG, Eisenhauer EA (2000). New guidelines to evaluate the response to treatment in solid tumors. European Organization for Research and Treatment of Cancer, National Cancer Institute of the United States, National Cancer Institute of Canada. J Natl Cancer Inst.

[15] Gehan EA, Tefft MC (2000). Will there be resistance to the RECIST (Response Evaluation Criteria in Solid Tumors)?. J Natl Cancer Inst.

[16] Tuma RS (2006). Sometimes size doesn’t matter: reevaluating RECIST and tumor response rate endpoints. J Natl Cancer Inst.

[17] Ratain MJ, Eckhardt SG (2004). Phase II studies of modern drugs directed against new targets: if you are fazed, too, then resist RECIST. J Clin Oncol.

[18] Eisenhauer EA, Therasse P, Bogaerts J (2009). New response evaluation criteria in solid tumours: revised RECIST guideline (version 1.1). Eur J Cancer.

[19] Fuse N, Nagahisa-Oku E, Doi T (2012). Effect of RECIST revision on classification of target lesions and overall response in advanced gastric cancer patients. Gastric Cancer.

[20] Nishino M, Jackman DM, Hatabu H (2010). New Response Evaluation Criteria in Solid Tumors (RECIST) guidelines for advanced non-small cell lung cancer: comparison with original RECIST and impact on assessment of tumor response to targeted therapy. AJR Am J Roentgenol.

[21] Marotta V, Ramundo V, Camera L (2012). Sorafenib in advanced iodine-refractory differentiated thyroid cancer: efficacy, safety and exploratory analysis of role of serum thyroglobulin and FDG-PET. Clin Endocrinol (Oxf).

[22] Sun S, Schiller JH (2007). Angiogenesis inhibitors in the treatment of lung cancer. Crit Rev Oncol Hematol.

[23] Nishino M, Jagannathan JP, Ramaiya NH, Van den Abbeele AD (2010). Revised RECIST guideline version 1.1: What oncologists want to know and what radiologists need to know. AJR Am J Roentgenol.

[24] Schlumberger M, Berg G, Cohen O (2004). Follow-up of low-risk patients with differentiated thyroid carcinoma: a European perspective. Eur J Endocrinol.

[25] Clark PM, Beckett G (2002). Can we measure serum thyroglobulin?. Ann Clin Biochem.

[26] Spencer CA, Takeuchi M, Kazarosyan M (1996). Current status and performance goals for serum thyroglobulin assays. Clin Chem.

[27] Erasmus JJ, Gladish GW, Broemeling L (2003). Interobserver and intraobserver variability in measurement of non-small-cell carcinoma lung lesions: implications for assessment of tumor response. J Clin Oncol.

[28] Bachelot A, Cailleux AF, Klain M (2002). Relationship between tumor burden and serum thyroglobulin level in patients with papillary and follicular thyroid carcinoma. Thyroid.

[29] Demers LM, Spencer CA (2003). Laboratory medicine practice guidelines: laboratory support for the diagnosis and monitoring of thyroid disease. Clin Endocrinol (Oxf).

[30] Mozley PD, Bendtsen C, Zhao B (2012). Measurement of tumor volumes improves RECIST-based response assessments in advanced lung cancer. Transl Oncol.

[31] Brose MS, Nutting CM, Sherman SI (2011). Rationale and design of decision: a double-blind, randomized, placebo-controlled phase III trial evaluating the efficacy and safety of sorafenib in patients with locally advanced or metastatic radioactive iodine (RAI)-refractory, differentiated thyroid cancer. BMC Cancer.

